# Next-generation sequencing and immuno-informatics for designing a multi-epitope vaccine against HSV-1-induced uveitis

**DOI:** 10.3389/fimmu.2025.1461725

**Published:** 2025-01-31

**Authors:** He Cao, Zhi Cao, Yue Han, Jing Shan

**Affiliations:** ^1^ Department of Ophthalmology, Shenzhen People’s Hospital (The Second Clinical Medical College, Jinan University; The First Affiliated Hospital, Southern University of Science and Technology), Shenzhen, Guangdong, China; ^2^ VPL Department, Mentor Graphics Technology (Shenzhen) CO. LTD., Shenzhen, Guangdong, China; ^3^ Nangang Branch, The Second Hospital of Heilong Jiang Province, Harbin, Heilongjiang, China; ^4^ The First Affiliated Hospital of Jiamusi University, Jiamusi, Heilongjiang, China

**Keywords:** epitopes, glycoproteins, herpes simplex virus type-1, NGS-immuno-informatics, uveitis

## Abstract

**Background:**

Uveitis, characterized by intraocular inflammation, poses significant clinical challenges, often leading to vision impairment or blindness. Herpes Simplex Virus type 1 (HSV-1) is a major cause of virus-induced uveitis. This study aims to design a novel multi-epitope vaccine targeting HSV-1 glycoproteins B, C, D, H, and L using an immuno-informatics approach, which are essential for viral entry and pathogenesis.

**Methods:**

The study identified epitopes for CD8+ T cells, CD4+ T cells, and B cells within the target glycoproteins. These epitopes were systematically evaluated for conservancy, immunogenicity, non-allergenicity, non-glycosylated regions, and binding affinities. A multi-epitope construct was designed, incorporating these epitopes along with an adjuvant, a PADRE sequence, and suitable linkers. In-silico immune simulations were performed to evaluate the vaccine’s potential to activate both innate and adaptive immune responses. Molecular docking simulations assessed the binding interactions between the multi-epitope vaccine and Toll-like receptor (TLR-9).

**Results:**

The selected epitopes demonstrated high conservancy, immunogenicity, and non-allergenicity. The multi-epitope construct effectively activated cytokine production, immunoglobulin secretion, and T cell responses in in-silico immune simulations. Molecular docking simulations showed strong binding interactions between the vaccine and TLR-9, suggesting enhanced antigen presentation capabilities.

**Conclusion:**

This comprehensive immuno-informatics approach provides a precision immunotherapy strategy for uveitis by leveraging computational modeling and predictive analytics to design a multi-epitope vaccine for HSV-1. The in-silico results indicate the vaccine’s potential efficacy in activating immune responses. Future experimental validation and clinical studies are necessary to confirm the safety and efficacy of this proposed vaccine in managing uveitis and preserving vision.

## 1 Introduction

**Figure 1:**
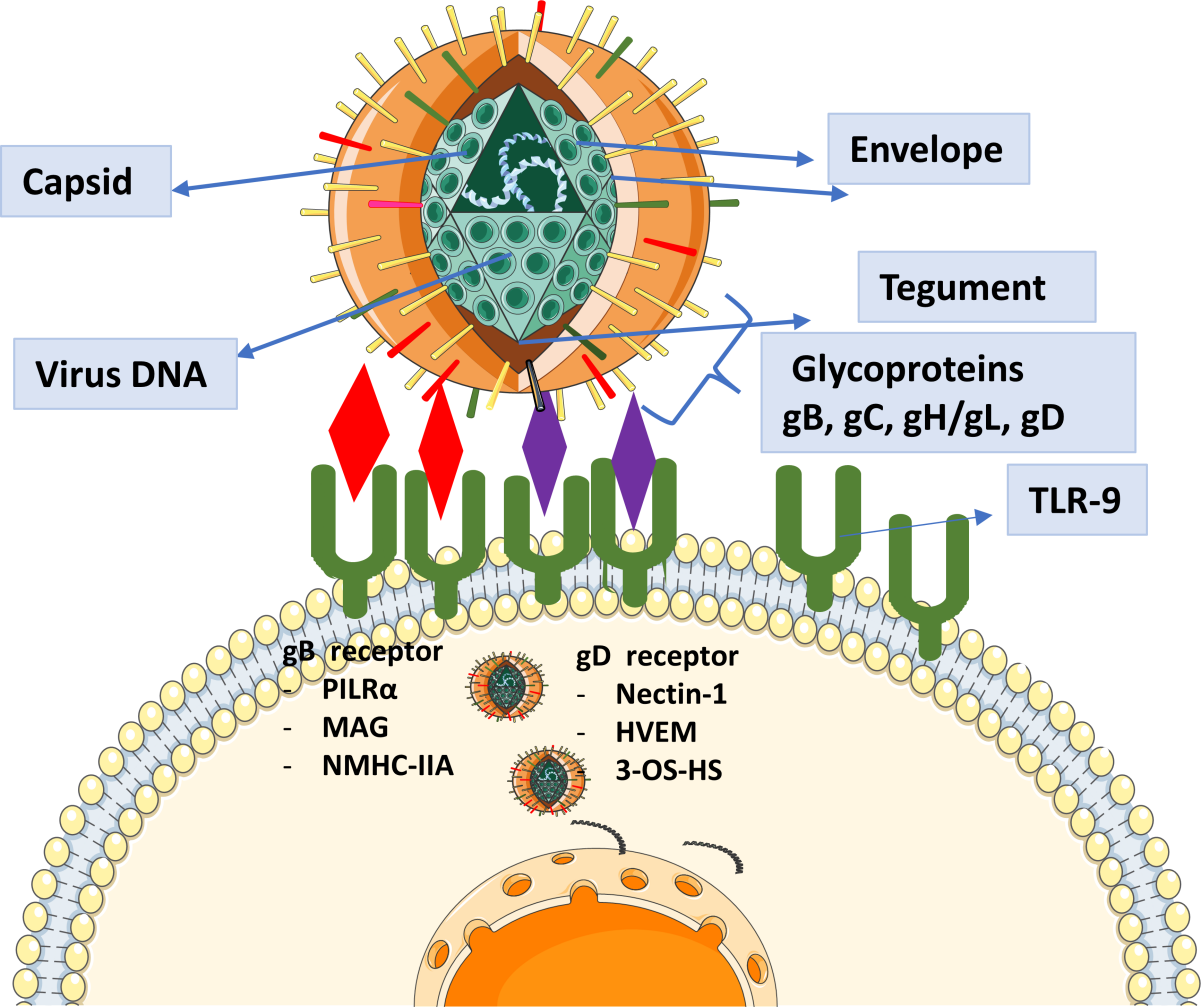
Representation of the role of glycoproteins in HSV-1 entry and their known receptors. Glycoproteins facilitate viral attachment, membrane fusion, and entry into host cells by interacting with specific cellular receptors. These interactions are critical for HSV-1 pathogenesis, enabling the virus to initiate infection and establish latency.

HSV-1 is a significant pathogen known for causing a wide range of infections, including ocular infections, alongside its more common oral and genital manifestations (1). This virus typically affects individuals in their fourth and fifth decades of life, irrespective of gender (2). HSV-1-associated ocular manifestations can range from primary infections of the ocular skin and mucosa, such as epidemic keratoconjunctivitis and keratouveitis, to persistent and recurrent intraocular infections, notably herpetic retinitis, often referred to as herpes simplex virus-1 retinitis (HSV-1R) (3). Initial HSV-1 infection typically happens through the oropharyngeal mucosa following exposure to secretions from a person shedding the virus. During the initial infection site, HSV-1 enters epithelial cells, undergoes replication, and subsequently travels in a retrograde manner through neurons to reach the dorsal root ganglia of the trigeminal nerve, where it establishes latency (4, 5). Ocular infection with HSV-1 is a leading cause of infectious blindness and accounts for most cases of herpes viral anterior and posterior uveitis. Reactivation of HSV-1 can lead to necrosis and inflammation of the iris, inflammation of the anterior chamber, and corneal stromal neovascularization, with anterior uveitis being more common during reactivation than during primary infection (6). HSV-1 can affect various ocular structures, resulting in clinical manifestations of differing severity (7). The spectrum of HSV-1-related ocular infections includes mild keratitis, necrotizing retinitis, acute retinal necrosis, immune-mediated endotheliitis, keratouveitis, and metaherpetic keratitis (8). The global prevalence of HSV-1 infection is concerning, with a substantial portion of the population hosting the virus in a latent state, ready for reactivation under favorable conditions. In 2016, the prevalence of HSV-1 among individuals aged 15-49 was recorded at 66.6% globally, with HSV-2 infection found in 13.2% of the population (9). Current antiviral treatments recommended by the World Health Organization (WHO) include aciclovir, valaciclovir, famciclovir, foscarnet, and cidofovir (10). However, there are significant concerns about drug resistance in immunocompromised individuals (11, 12), and current treatments suffer from limited selectivity, bioavailability, and significant renal toxicity (13). Given the high prevalence of HSV-1 infections and the increasing resistance to existing treatments, developing new approaches for prevention and management is crucial. The mature HSV-1 virus particle contains a double-stranded DNA genome exceeding 150 kilobase pairs, enclosed in an icosahedral protein capsid. This capsid is surrounded by a tegument layer mainly consisting of viral proteins, which is in turn enveloped by a lipid bilayer membrane. The viral envelope contains glycoproteins encoded by the virus, such as gB, gC, gD, gH, and gL, which play crucial roles in facilitating viral entry into host cells (14, **Figure 1**). HSV-1 envelope glycoproteins bind to specific receptors on the cell surface, facilitating membrane fusion and allowing the viral nucleocapsid to enter the cytoplasm. This entry process can take place either directly at the cell surface or after uptake into an intracellular vesicle through endocytosis or phagocytosis. Central to the pathogenesis of HSV-1 is the intricate interplay of viral glycoproteins during host cell entry. These glycoproteins, particularly gB, gD, and gH/gL, facilitate viral attachment, fusion, and penetration into host cells, establishing infection (15). Understanding the pivotal role of these glycoproteins not only illuminates the viral pathogenesis but also holds promise for targeted therapeutic interventions aimed at disrupting crucial viral entry mechanisms. Our study employs an innovative immuno-informatics-driven approach to design a novel vaccine targeting HSV-1-associated uveitis. By leveraging computational modeling and predictive analytics, we aim to develop immunotherapeutic agent that can effectively combat the burden posed by HSV-1-induced ocular diseases.

## 2 Methodology

### 2.1 Selection of glycoproteins

The protein sequences of HSV-1 glycoproteins B, C, D, H, and L were obtained from the UNIPROT database. These sequences were subjected to NCBI-BLAST analysis, from which approximately 250 sequences with varied variabilities were randomly selected for each glycoprotein. Multiple sequence alignments were performed to identify variable regions within each glycoprotein. The analysis of the physicochemical properties of glycoproteins was performed employing the PROTPARAM tool (16).

### 2.2 Epitope prediction

The Tepitool from the IEDB was used to screen for CTL and HTL epitopes within each glycoprotein (17). Epitopes were predicted for the four most widely spread alleles: HLA Class II alleles: DRB1*01:01 and DQB1*03:01; HLA Class I alleles: A*02:01 and B*07:02. 9-mers were used for predicting epitopes for HLA Class I, and 15-mers were used for HLA Class II epitopes. We utilized the Bepipred 2.0 tool with a threshold of 0.5 to predict linear B cell epitopes (18).

### 2.3 Epitope screening

Predicted epitopes were further filtered based on binding affinity (as revealed by the Tepitool server), antigenicity, non-allergenicity, conservancy, and location in non-glycosylated regions.

### 2.4 Molecular docking

The filtered epitopes were subjected to molecular docking analysis using the Cluspro server (19). We performed docking of HLA Class II epitopes with the HLA-DRB101:01 allele, and HLA Class I epitopes were docked with the HLA-A02:01 allele. It was conducted to analyze the interaction patterns of epitopes and HLA alleles and to further filter them based on their interaction affinities and patterns.

### 2.5 Developing multi-epitope vaccine and docking

The selected epitopes were assembled into a multi-epitope construct linked by appropriate linkers. An adjuvant (cytidine phosphate guanosine (CpG) oligodeoxynucleotides (ODN)) and a PADRE sequence were included to enhance the immunogenicity of the vaccine. The vaccine construct was submitted to Tepitool server to investigate if it includes epitopes targeting other HLA alleles. In addition, population coverage of the vaccine sequence was analyzed based on the interacting HLA Alleles as revealed by Tepitool server, using the IEDB population coverage tool. The vaccine sequence was modeled using the I-TASSER server (20) and refined with the GalaxyRefine server (21). The structuaral quality of the final vaccine structure was evaluated using Ramachandran plot analysis via the MolProbity server (22). The flexibility of the vaccine structure was examined using online simulation tool CABSFlex 2.0 (23).

### 2.6 Molecular docking of vaccine model and TLR-9

Both the 3D modeled vaccine and TLR-9 underwent molecular docking analysis to evaluate the affinity, stability and efficacy of the vaccine-receptor interactions. Cluspro server was employed for interaction analysis.

### 2.7 *In silico* immune simulation analysis

The potential immune response that may be elicited by the vaccine was analyzed through *in silico* immune simulations, focusing on the activation of the cell components belonging to innate and active immune responses, by assessing cytokine, immunoglobulin, and T cell responses. ImmSim server was employed for the same (24).

## 3 Results and discussion

### 3.1 HSV-1 glycoproteins

**Figure 2:**
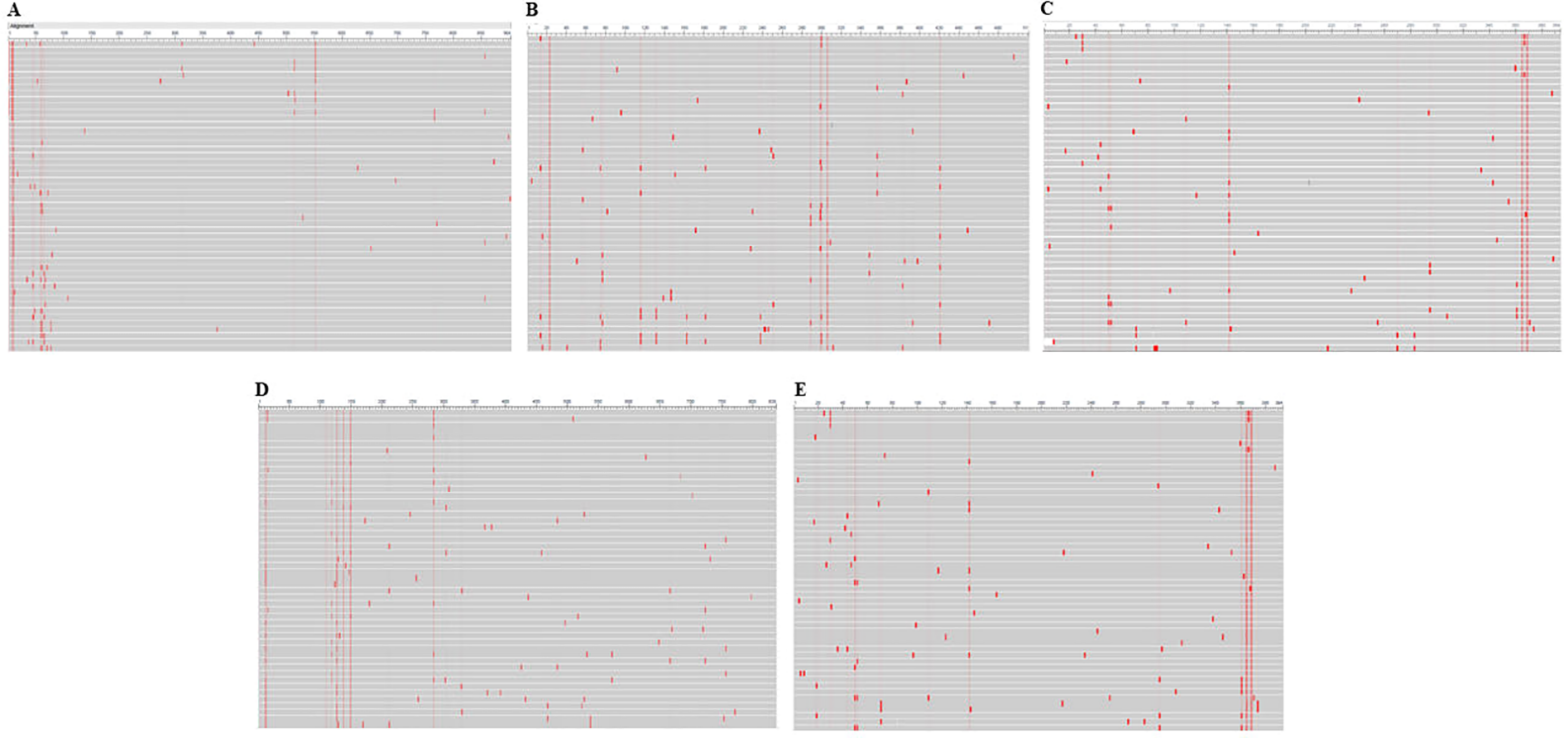
Conservancy analysis of selected glycoproteins using the COBALT tool. Panels **(A–E)** represent the conservation patterns in glycoproteins B, C, D, H, and L, respectively. Red regions indicate sequence alterations, highlighting variability critical to understanding immune escape mechanisms.

The amino acid sequences of HSV-1 glycoproteins were aligned using the Cobalt tool to investigate potential mutations. COBALT is a Constraint-based Multiple Alignment Tool used to perform multiple sequence alignment. In this study, we employed COBALT to identify conserved regions and highlight mutations in glycoprotein sequences. Mutations were visualized using a frequency-based distribution approach, allowing us to map areas of alteration effectively (**Figure 2**). Identified mutations led to the omission of predicted epitopes located within these regions. Furthermore, the physicochemical properties of these glycoprotein were assessed using the ProtParam tool (**Supplementary Table 1**). This approach is consistent with previous immuno-informatics studies, such as the work by Chauhan et al., who predicted epitopes targeting the glycoproteins of Cytomegalovirus, which facilitate its entry into the host (25).

### 3.2 Epitope mapping and screening

**Figure 3:**
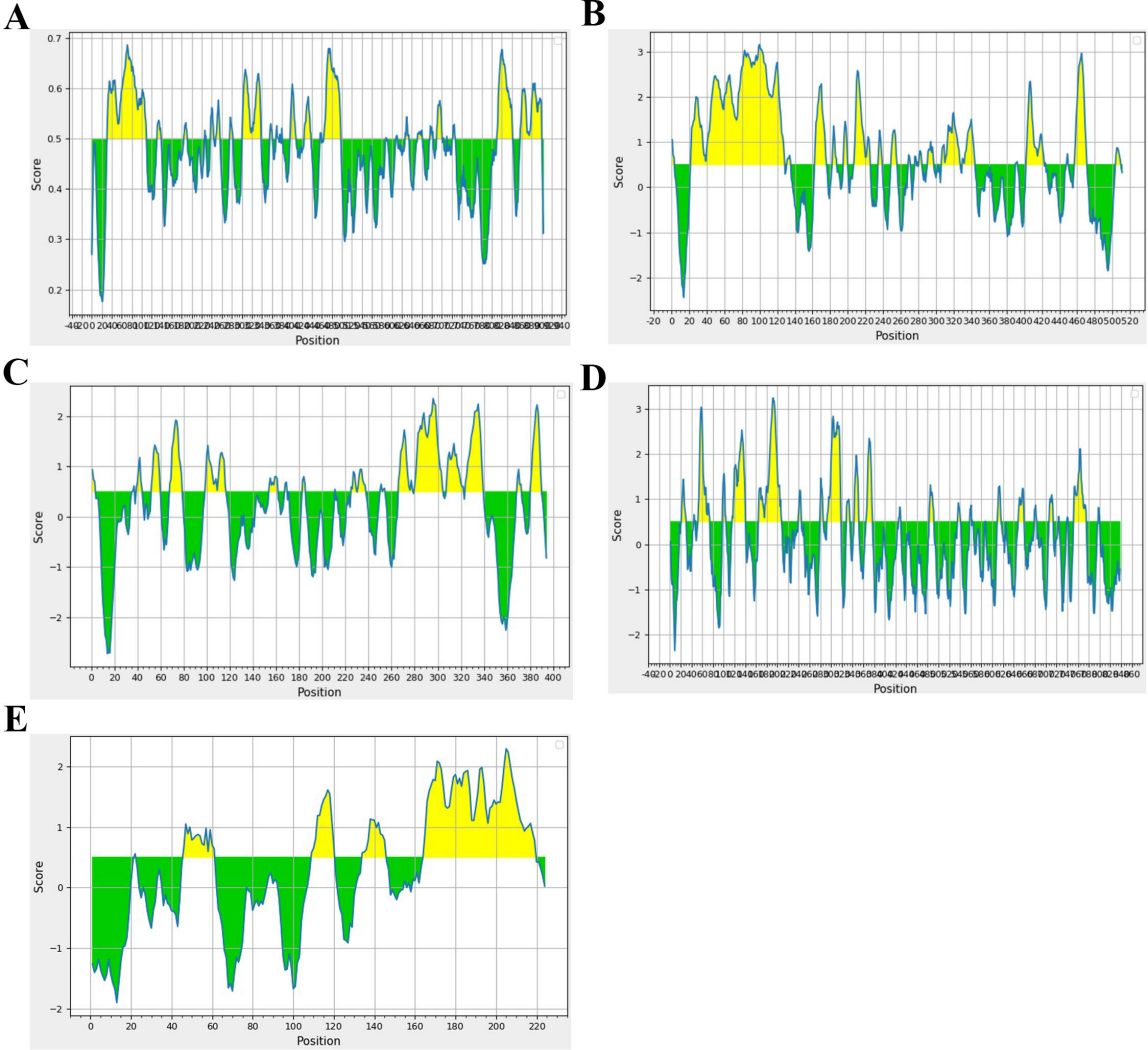
Spanning of B cell epitopes in each glycoprotein. **(A)** Glycoprotein B, **(B)** Glycoprotein C, **(C)** Glycoprotein D, **(D)** Glycoprotein H, and **(E)** Glycoprotein L.

Epitopes for MHC Class I and II T cells were predicted using the IEDB-Tepitool server. HLA-A02:01 and HLA-B07:02 restricted CTL epitopes were identified without altering any parameters, using 9-mer peptides. Duplicate epitopes were automatically removed by the tool, and peptides with a predicted consensus percentile rank of ≤1 were selected for further screening. Epitopes for B cells were predicted using Bepipred-2.0 tool with a threshold set to 0.5 by default (**Figure 3**). Initially, the T cell epitopes predicted by the Tepitool server were checked for their location in non-mutational sites. Subsequently, these epitopes were evaluated for their immunogenicity/antigenicity scores, as peptides typically exhibit low antigenicity, making immunogenicity screening crucial. Immunogenic epitopes were then extracted. The allergenic potential of the epitopes was evaluated, and only those identified as non-allergenic were included. Additionally, the epitopes’ locations were checked for the presence of glycosylated regions (**Supplementary Figure 1**) and those within glycosylated regions were omitted. The remaining epitopes were subjected to molecular docking analysis, focusing on their binding patterns and affinities.

For molecular docking, HTL and CTL epitopes were docked with the DRB1*01:01 A*02:01 allele, respectively as the primary goal was to evaluate binding patterns and affinities. After this rigorous filtering process, 11-CTL, 13-HTL, and 5 B-cell epitopes were finalized (**Table 1**). Similar strategies for filtering potential epitopes for vaccine purposes have been employed in previous studies targeting various pathogens, including viruses (25), bacteria (26), parasitic diseases (27), and metabolic diseases like cancer (28).

**Table 1:** ^*^Class I epitopes, ^#^Class II epitopes and ^**^B cell epitopes finalized in each glycoprotein.

Position	Peptide sequence	Peptide having affinity toward HLA allele
Glycoprotein B		
287-295	FVLATGDFV^*^	A02
777-785	ALAVGLLVL^*^	A02
116-124	CPPPTGATV^*^	B07
55-69	APPPLGAAPTGDPKP^#^	HLA-DQA1*05:01/DQB1*03:01
229-243	ETDMELKPANAATRT^#^	DRB1
284-298	YDEFVLATGDFVYMS^#^	DRB1
778-792	LAVGLLVLAGLAAAF^#^	DRB1
223-249	FHRDDHETDMELKPANAATRTSRGWHT^**^	
Glycoprotein C		
423-431	GLATVRSTL^*^	A02, B07
404-418	DPSPAAKSAVTAQES^#^	HLA-DQA1*05:01/DQB1*03:01
480-494	GIGIGVLAAGVLVVT^#^	DRB1
306-344	TVTSEAVGGQVPPRT^**^	
Glycoprotein D		
61-69	RVYHIQAGL^*^	A02, B07
248-256	FIPENQRTV^*^	A02
120134	LTIAWFRMGGNCAIP^#^	DRB1
174-178	LGFLMHAPAFETAGT^#^	DRB1
225-238	SACLSPQAYQQGVT^**^	
Glycoprotein H		
790-798	LLAFDTQPV^*^	A02
810-818	ALGVVMITA^*^	A02
587-595	SPCAASLRF^*^	B07
231-245	PGRYVYFSPSASTWP^#^	HLA-DQA1*05:01/DQB1*03:01
376-390	PPLFWRLTGLLATSG^#^	DRB1
809-823	SALGVVMITAALAGI^#^	DRB1
365-377	SGDAGAEQGPRPP^**^	
Glycoprotein L		
186-194	GLQPKPLTT^*^	A02
58-66	TPSAINYAL^*^	B07
142-156	HTPVKAGCVNFDYSR^#^	DRB1
195-209	PPPIIATSDPTPRRD^#^	DRB1
`164-221	QDLGPTNGTSGRTPVLPPDDEAGLQPKPLTTPPPIIATSDPTPRRDAATKSRRRRPHS^**^	

### 3.3 Multi-epitope vaccine

**Figure 4:**
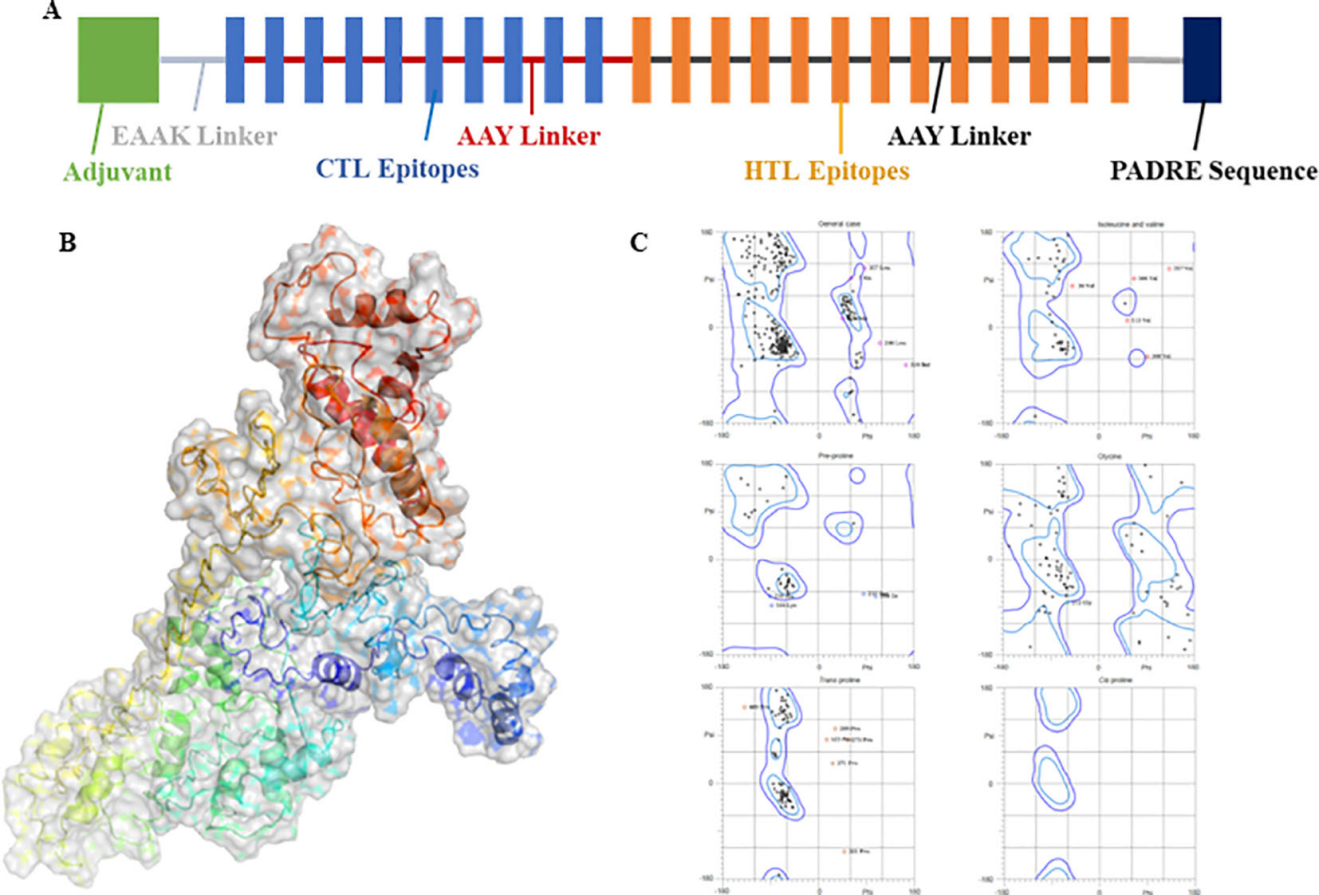
**(A)** A strategic layout of the vaccine designed. **(B)** 3D modeled protein of vaccine using I-Tasser server. **(C)** Ramachandran plot analysis of the refined 3D modeled protein.

**Figure 5:**
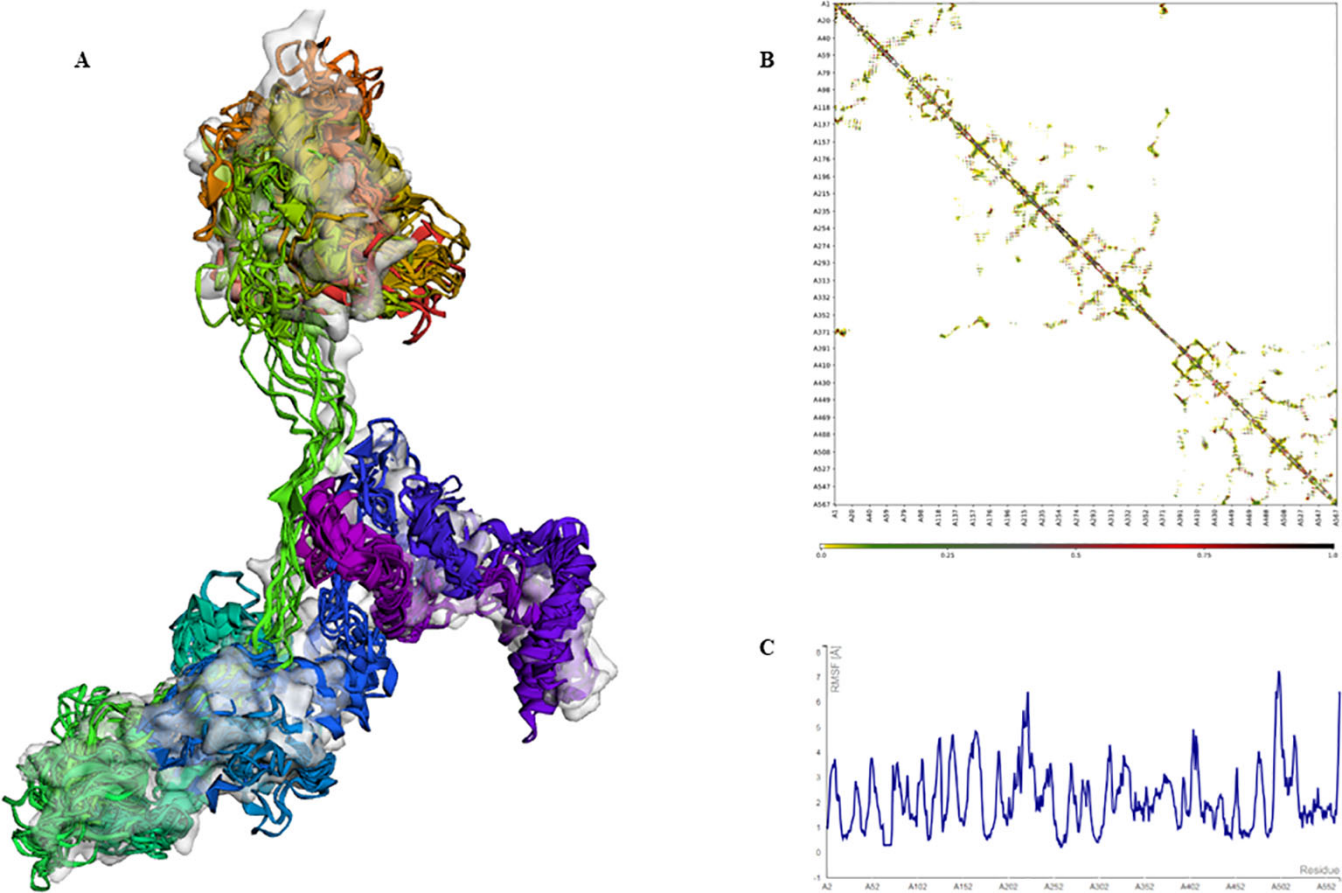
Simulation of the 3D modeled vaccine for flexibility analysis. **(A)** A cartoon and surface depiction of the 10-final models, illustrating limited overall fluctuations, with minor variations in the red and brown regions where the adjuvant is situated. **(B)** Contact-map showing residue-residue interactions within the protein, with interactive regions highlighted in the central panel. Notably, there are pronounced interaction patterns in the latter part of the sequence where the epitopes are located. **(C)** The fluctuation plot illustrates residue movements during the simulation, indicating enhanced flexibility in the latter segment of the sequence.

The extracted epitopes were meticulously assembled into a cohesive epitope chain. Each epitope was linked using specific linkers: AAY, GPGPG, and KK. To enhance the immunogenicity, an adjuvant CpG-ODN and a PADRE sequence were incorporated into the chain via an EAAAK linker (**Figure 4A**). The constructed epitope chain was then subjected to homology modeling to obtain a 3D structure. Using the I-TASSER server, the 3D model of the vaccine was generated, yielding a TM-score, C-score and RMSD as 0.39 ± 0.13, -2.86, 14.7 ± 3.6Å, respectively (**Figure 4B**). These scores indicate the predicted quality and reliability of the model, with the TM-score suggesting moderate structural similarity to known proteins. Following this, the initial 3D model underwent refinement using the Galaxy-Refine server to enhance its structural accuracy. The refined model’s quality was assessed using Ramachandran plot analysis, revealing that 96.3% of the amino acids were located in favored regions, indicating a well-modeled structure (**Figure 4C**). Further analysis included simulation studies to assess both the flexibility and stability of the vaccine construct. Ten 3D models were assessed, showing minor structural adjustments at the beginning and significant changes in the latter part where epitopes were linked. Fluctuations ranging from 0Å to 7Å were observed in the regions containing epitopes, suggesting enhanced flexibility in these segments (**Figures 5A–C**). This flexibility is crucial for the vaccine’s interaction with the immune system, potentially improving antigen presentation and immune response. The methodological approach and the resulting data align with previous studies, which have employed similar strategies for vaccine modeling and validation. For instance, Chauhan et al., (25) utilized a comparable framework to develop epitope-based vaccines targeting various pathogens, demonstrating the robustness and applicability of this technique.

### 3.4 Interaction analysis of vaccine and TLR-9

**Figure 6:**
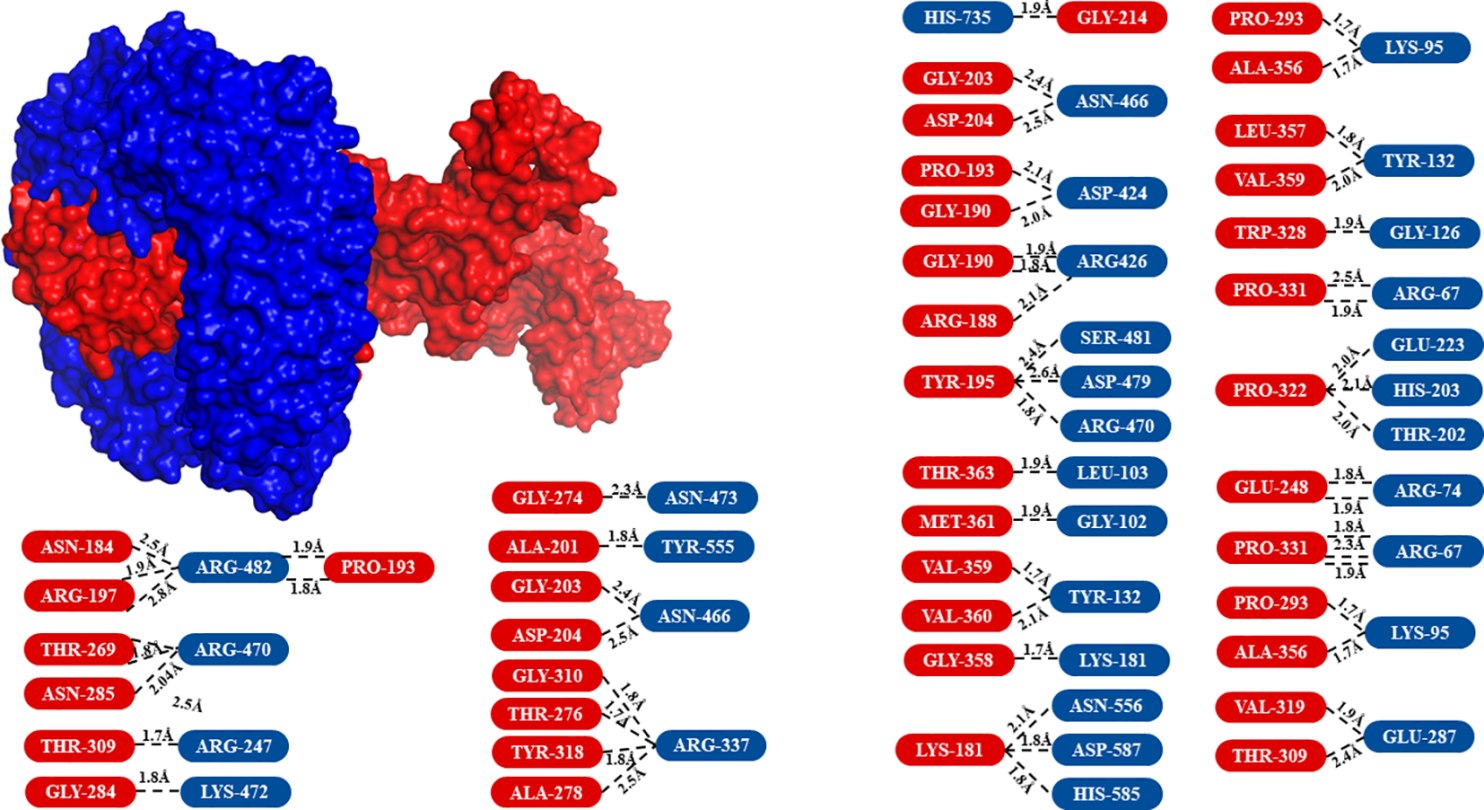
Surface view of the molecular docking analysis between the vaccine and TLR-9. The receptor, TLR-9, is shown in blue, while the vaccine (ligand) is displayed in red. The amino acids involved in the interaction are highlighted in the same colors as their respective 3D model representations.

The modeled vaccine was subjected to molecular docking analysis with TLR-9 to evaluate the potential interactions and binding affinity. The 3D structure of TLR-9 was acquired from the Protein Data Bank under the ID 3WPF. Prior to docking, heteroatoms, including water molecules and any attached ligands, were removed from the TLR-9 3D model to ensure accurate interaction analysis. TLR-9 (Toll-like receptor 9) is a pattern recognition receptor that plays a critical role in innate immunity. It recognizes unmethylated CpG motifs present in bacterial and viral DNA, leading to the activation of downstream signaling pathways and the production of pro-inflammatory cytokines. The molecular docking analysis was conducted using the ClusPro server, which provided insights into the binding interactions between the vaccine and TLR-9. The results demonstrated that the vaccine had a high affinity for TLR-9, binding effectively within its pocket and forming multiple hydrogen bonds (**Figure 6**). These interactions suggest a stable and robust binding, which is crucial for the vaccine’s potential to stimulate an immune response through TLR-9-mediated signaling pathways. The vaccine’s strong binding affinity to TLR-9 is indicative of its potential efficacy in enhancing antigen presentation and triggering a strong immune response. This interaction is crucial for triggering the innate immune system, which can subsequently activate adaptive immunity, thereby offering a comprehensive defense against HSV-1. The docking results underscore the importance of the selected epitopes and the effectiveness of the multi-epitope construct design. Ensured effective binding to TLR-9, the vaccine is likely to facilitate the recognition and processing of viral antigens, thereby improving its immunogenic potential. This finding is consistent with previous studies that have shown the importance of TLR-9 interactions in the effectiveness of vaccine constructs (25).

### 3.5 Computational immune simulation

**Figure 7:**
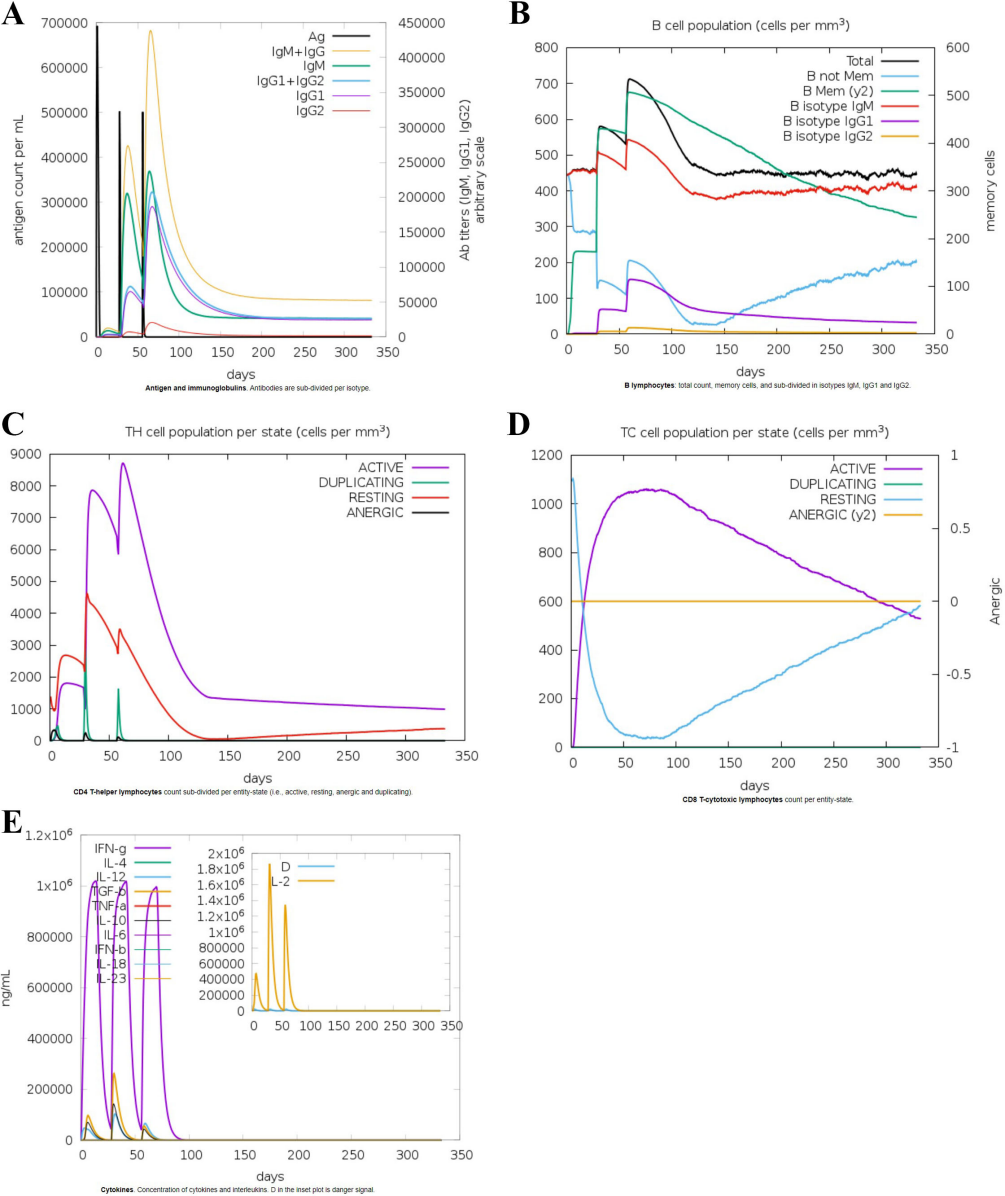
*In-silico* immune simulation of the vaccine sequence: **(A)** Antigen and immunoglobulin responses, **(B)** B lymphocytes response, **(C)** CD-4 T helper lymphocyte response, **(D)** CD-8 T cytotoxic lymphocyte count, **(E)** Interleukins response.

To assess the immunogenic potential of the multi-epitope vaccine, computational immune simulations were performed using the C-ImmSim server. The simulation parameters included a simulation volume set to 25, with the number of steps fixed at 1000. Three injections were administered at intervals of 0, 84, and 168 days to mimic booster vaccinations. The simulation results demonstrated that the vaccine sequence significantly enhanced immunogenicity. Key observations included elevated levels of cytokines, which are critical mediators of immune responses. Additionally, there was a notable increase in the population of cytotoxic T cells, indicating a strong cellular immune response capable of targeting and eliminating infected cells. The formation of memory B cells was also significantly enhanced. This aspect is pivotal for long-term immunity, as memory B cells empower the immune system to mount quicker and more effective responses upon subsequent exposures to the antigen. The presence of robust memory B cell populations suggests that the vaccine could provide sustained protection against HSV-1. These findings are illustrated in **Figure 7**, which shows the temporal dynamics of cytokine levels, cytotoxic T cell responses, and memory B cell formation over the course of the simulation. The heightened immunogenicity observed in the computational simulation underscores the potential effectiveness of the multi-epitope vaccine in eliciting strong and durable immune responses. By stimulating both innate and adaptive immune pathways, the vaccine is poised to offer comprehensive protection against HSV-1-induced uveitis. The credibility of *in silico* immune simulations is supported by studies where computationally predicted immunogenic vaccines have demonstrated promising results in experimental validation. For instance, a recent study by Jiang et al. reported the successful *in vitro* testing of a multi-epitope vaccine designed through in silico methods. The immunogenic potential of the vaccine was initially assessed using the ImmSim server, highlighting the reliability of such computational approaches in vaccine development (29).

## 4 Conclusion

This study presents a comprehensive immuno-informatics-based approach to develop a vaccine comprising of a series of epitopes targeting HSV-1 glycoproteins for uveitis immunotherapy. By identifying and rigorously screening CTL, HTL, and B cell epitopes, a robust peptide construct was developed. Molecular docking showed substantial binding between the vaccine and TLR-9, implying effective antigen presentation. *In-silico* immune simulations demonstrated enhanced immunogenicity, with elevated cytokine levels, cytotoxic T cell activity, and memory B cell formation. These promising results provide a solid foundation for future experimental validation and clinical evaluation, potentially offering a novel and effective strategy for managing HSV-1-induced uveitis and preserving vision.

## Data Availability

The datasets presented in this study can be found in online repositories. The names of the repository/repositories and accession number(s) can be found in the article/**Supplementary Material**.
