# Supplementary material for: Next-generation sequencing and immuno-informatics for designing a multi-epitope vaccine against HSV-1-induced uveitis
Source: Front Immunol. 2025 Jan 31;16:1461725. doi: 10.3389/fimmu.2025.1461725 (PMC11825787; doi:10.3389/fimmu.2025.1461725)
Supplement: Supplementary file 1 [file DataSheet1.docx]

| **Glycoproteins** | **Molecular weight** | **Theoretical pI** | **Extinction coefficient M^-1^ cm-^1^, at 280 nm** | **Estimated half-life (mammalian reticulocytes, in vitro)** | **Instability Index** | **Aliphatic index** | **Grand average of hydropathicity (GRAVY)** |
| --- | --- | --- | --- | --- | --- | --- | --- |
| gB | 100368.44 | 7.63 | 110755 | 30 hours | 39.75 | 70.93 | -0.398 |
| gC | 55011.16 | 7.65 | 81860 | 30 hours | 46.89 | 67.36 | -0.353 |
| gD | 43346.88 | 7.64 | 58330 | 30 hours | 61.11 | 89.42 | -0.143 |
| gH | 90366.24 | 6.61 | 112800 | 30 hours | 42.65 | 92.85 | 0.052 |
| gL | 24933.53 | 9.32 | 33920 | 30 hours | 54.27 | 84.46 | -0.355 |

**Supplementary table 1:** Physicochemical properties of HSV-1 Glycoproteins: B, C, D, H and L


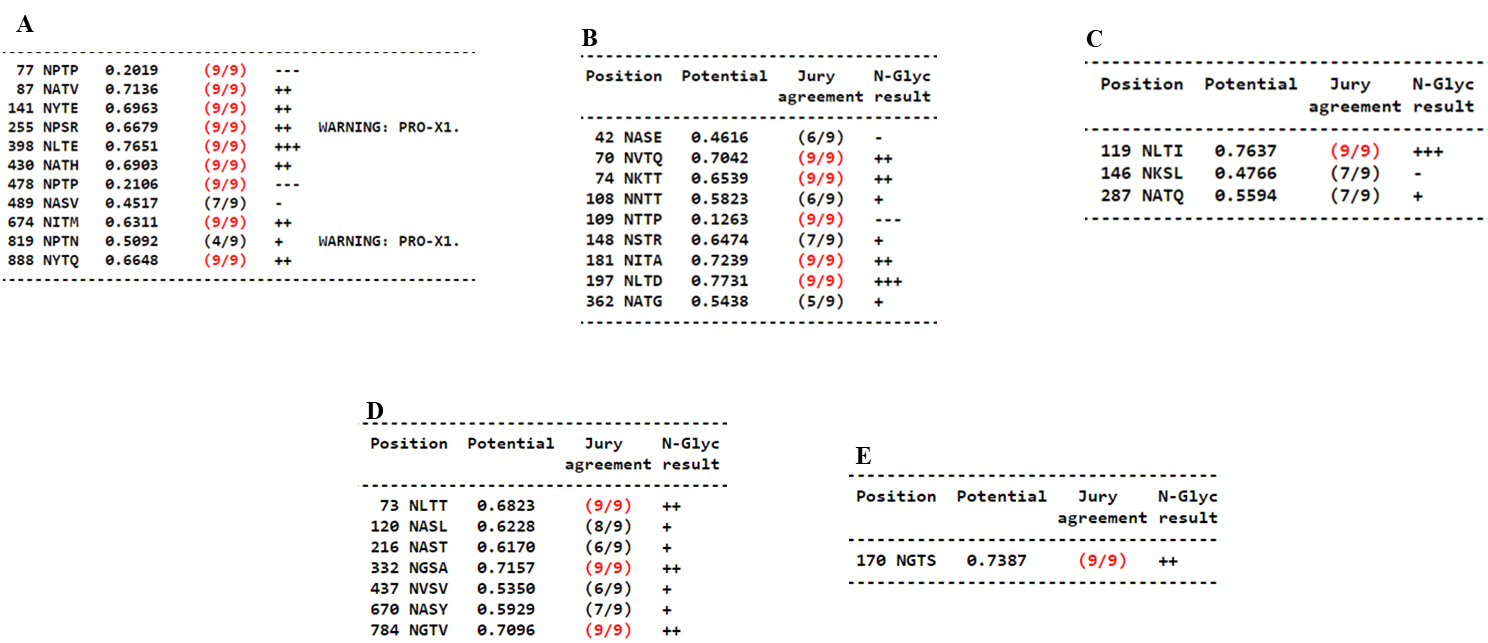


**Supplementary Figure 1:** Glycosylated regions predicted in each glycoprotein. (A): glycoprotein B; (B): glycoprotein C; (C) glycoprotein D; (D) glycoprotein H and (E) glycoprotein L.
